# Zinc Complexes Containing Coumarin-Derived Anilido-Aldimine Ligands as Catalysts for Ring Opening Polymerization of l-Lactide

**DOI:** 10.3390/molecules20045313

**Published:** 2015-03-24

**Authors:** Chi-Tien Chen, Min-Chian Wang, Tzu-Lun Huang

**Affiliations:** Department of Chemistry, National Chung Hsing University, Taichung 402, Taiwan; E-Mails: a23444452@gmail.com (M.-C.W.); s912218@gmail.com (T.-L.H.)

**Keywords:** coumarin-derived ligands, anilido-aldimine, zinc complexes, ring opening polymerization, l-lactide

## Abstract

The coumarin-derived ligand precursors **L^1^H**–**L^6^H** have been prepared. Treatment of these ligand precursors with 1.2 equiv. of ZnEt_2_ in toluene affords zinc ethyl complexes (LZnEt) **1**–**6** (where L = coumarin-derived ligands bearing different functional groups). Reaction of ligand precursor **L^3^H** with 1.5 equiv. of Zn[N(SiMe_3_)_2_]_2_ in toluene affords the zinc amide complex, L^3^ZnN(SiMe_3_)_2_, **7**. All these compounds were characterized by NMR spectroscopy and elemental analysis. The molecular structures are reported for **1** and **7**. The catalytic activities of complexes **1**–**7** towards the ring opening polymerization of l-lactide in the presence of 9-AnOH have been investigated.

## 1. Introduction

Due to the biodegradability, biocompatibility, and permeability properties demonstrated by polylactide, development of well-defined metal complexes involved in ring opening polymerization as catalysts/initiators has recently become attractive [1,2,3,4]. Among these studies, metal complexes bearing nitrogen-based ligands are extensively applied, and their structures and chemistry have been reviewed [5,6,7,8,9,10,11]. The promising activities and controlled behavior demonstrated by those complexes have encouraged diverse research groups to synthesize similar chelating ligand precursors and examine the bonding and electronic features related to these ligands. Recently some metal complexes containing anilido-aldimine ligands have been reported and demonstrated catalytic activities in ring opening polymerization of cyclic esters [12,13,14,15,16,17,18,19]. New ligand precursors working with similar chelating systems should be the attractive candidates. Therefore we introduce similar bonding mode as found in the anilido-aldimine ligand into coumarin molecules whose derivatives demonstrate biological activity [20,21,22,23], or are sensitive fluorescent probes [24] or organic dyes [25], to prepare several coumarin-derived anilido-aldimine ligand precursors. We expect those precursors to have the potential to work as mono anionic dentate ligands upon reacting with zinc reagents. The catalytic activities of these zinc complexes towards the ring opening polymerization of l-lactide in the presence of alcohols have also been investigated.

## 2. Results and Discussion

### 2.1. Syntheses and Characterization of Ligand Precursors and Zinc Complexes

**Scheme 1:**
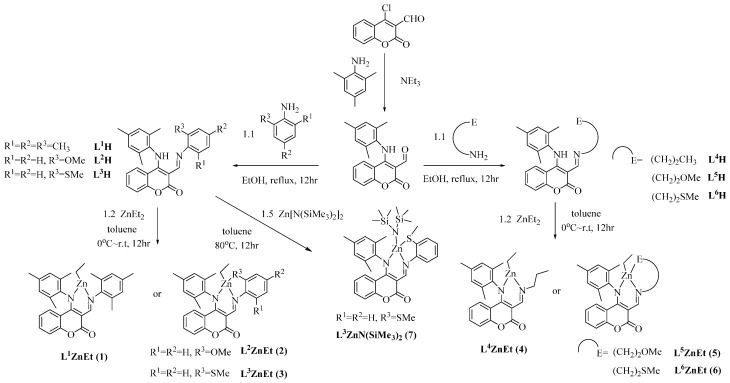
Preparation of ligand precursors and zinc complexes.

Ligand precursors **L^1^H**–**L^6^H** were prepared in a straightforward fashion by the condensation between 4-(mesitylamino)-2-oxo-2*H*-chromene-3-carbaldehyde and substituted anilines or aliphatic amines to afford the target compounds in fair to high yield. Due to the promising catalytic activities exhibited by zinc anilido-aldimine complexes in ring opening polymerization reactions [12,15,16,17,19], preparations of zinc ethyl complexes were examined by the reactions of **L^1^H**–**L^6^H** with ZnEt_2_ in a ratio of 1:1 in toluene solution resulting in the isolation of zinc ethyl complexes **1**–**6**. The zinc amide complex **7**, L^3^ZnN(SiMe_3_)_2_, was prepared by the reaction of **L^3^H** with Zn[N(SiMe_3_)_2_]_2_. The spectroscopic and elemental analysis data of **1**–**7** are consistent with the structures proposed in Scheme 1. Attempts to synthesize zinc benzyl oxide complexes have so far been proved unsuccessful.

**Figure 1:**
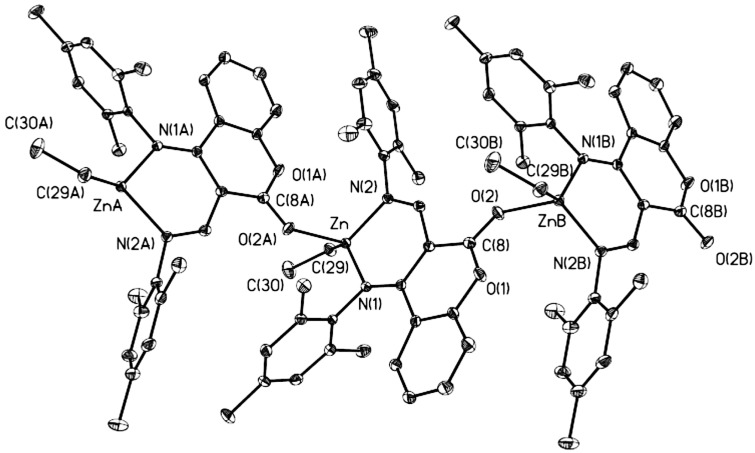
Molecular structure of **1**. A continuous 1D-array in **1** is achieved by coordination of carbonyl groups to metal centers.

**Figure 2:**
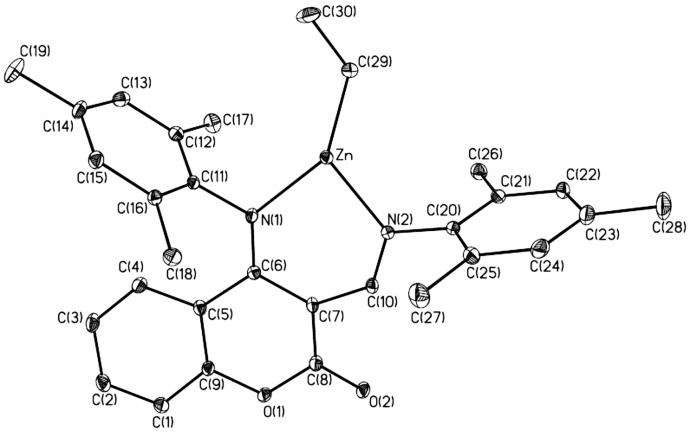
Molecular structure of **1** (hydrogen atoms omitted for clarity).

Suitable crystals for structural determination of **1** or **7** were obtained from toluene/hexane solution (compound **1**) or concentrated hexane solution (compound **7**). The molecular structure of **1** exists as a coordination polymer and the diagrams of its molecular structure are depicted in Figure 1 and Figure 2. Selected bond lengths and bond angles are summarized in Table 1.

**Table 1:** Selected bond lengths (Å) and bond angles (°) for **1** and **7**.

**1** *^a^*			
Zn-N(1)	2.0650(17)	N(1)-C(6)	1.310(3)
Zn-N(2)	2.0256(17)	N(2)-C(10)	1.290(3)
Zn-C(29)	1.987(2)	C(6)-C(7)	1.444(3)
Zn-O(2A)	2.1062(16)	C(7)-C(10)	1.437(3)
N(2)-Zn-N(1)	90.95(7)	C(29)-Zn-O(2A)	106.29(8)
C(29)-Zn-N(1)	132.97(9)	N(1)-Zn-O(2A)	92.54(6)
C(29)-Zn-N(2)	124.07(8)	N(2)-Zn-O(2A)	103.41(7)
**7** *^b^*			
Zn-N(1)	2.0314(19)	N(1)-C(6)	1.316(3)
Zn-N(2)	1.9932(19)	N(2)-C(10)	1.300(3)
Zn-N(3)	1.916(2)	C(6)-C(7)	1.442(3)
Zn-S	2.6488(6)	C(7)-C(10)	1.419(3)
N(2)-Zn-N(1)	92.30(8)	N(3)-Zn-S	106.68(6)
N(3)-Zn-N(2)	128.82(8)	N(2)-Zn-S	76.57(6)
N(3)-Zn-N(1)	120.25(8)	N(1)-Zn-S	125.91(5)

*^a^* Symmetry elements for 1: −x + 1/2, −y, z + 1/2; −x, y + 1/2, −z + 1/2; x + 1/2, −y + 1/2, −z; *^b^* Symmetry elements for 7: −x, −y, −z.

Each of the zinc atoms is four-coordinate, and is bonded to one nitrogen atom from the imino group with a Zn-N(1) bond distance of 2.0650(17)Å, one nitrogen atom from the anilido group with a Zn-N(2) bond distance of 2.0256(17)Å, one carbon atom from the ethyl group with a Zn-C(29) bond distance of 1.987(2)Å, and one oxygen atom from the carbonyl group of another molecule with a Zn-O(2) bond distance of 2.1062(16)Å. The coordination of another molecule via the carbonyl oxygen atom could be another evidence for the coordination-insertion mechanism of ring opening polymerization [26,27,28]. The geometry around the zinc centre of **1** can be described as a distorted tetrahedral geometry with N(2)-Zn-N(1) and C(29)-Zn-O(2) angles of 90.95(7)° and 106.29(8)°. These data are within the range of the known distances and angles for zinc anilido-aldimine complexes [12,15,16,17,19] and structurally-related zinc complexes [29,30].

**Figure 3:**
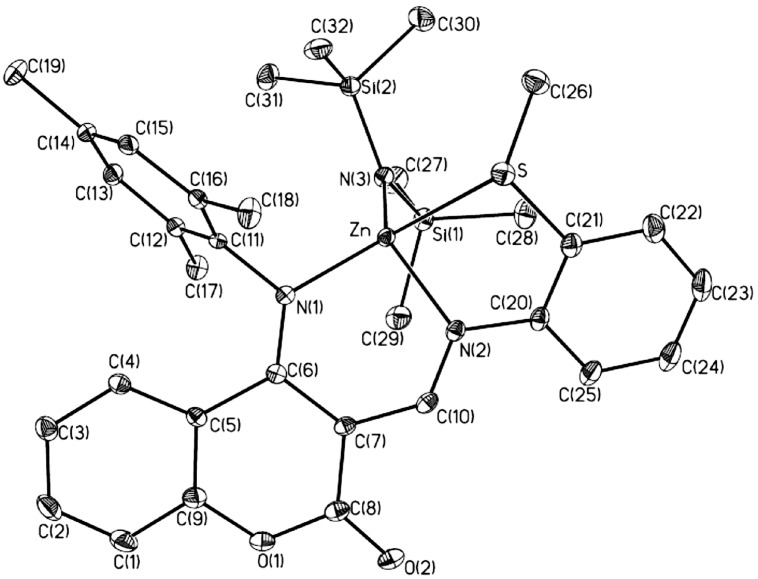
Molecular structure of **7**. Hydrogen atoms omitted for clarity.

Basically, compound **7** is quite similar to compound **1** albeit with a 2-methylthio substituent instead of 2,4,6-trimethyl substituents on the phenyl group of the imino part for **1** and the amide group instead of an ethyl group, as shown in Figure 3. Like **1**, the zinc atom is four-coordinate with one nitrogen atom from the imino group with a Zn-N(2) bond distance of 2.0314(19)Å, one nitrogen atom from the anilido group with a Zn-N(1) bond distance of 1.9932(19)Å, one nitrogen atom from the amide group with a Zn-N(3) bond distance of 1.916(2)Å, and one sulphur atom from the pendant functionality with a Zn-S bond distance of 2.6488(6)Å. The geometry around the zinc centre of **7** can be described as a distorted tetrahedral geometry with a smaller N(2)-Zn-S angle [76.57(6)°]. Bond lengths and bond angles are similar to those discussed above for **1**. The bond distance of the zinc amide is close to those found in the literature [31,32]. The Zn-S bond distance is a bit longer than that reported in the literature [33].

### 2.2. Polymerization Studies

Since several zinc anilido-aldimine complexes are known as efficient catalysts/initiators for the ring opening polymerization (ROP) of cyclic esters [12,15,16,17,19], the catalytic activities of structurally-related zinc ethyl complexes **1**–**6** towards the ROP of l-lactide were examined in the presence of 9-AnOH in toluene at 50 °C for 30 min. under a dry nitrogen atmosphere (entries 1–6). Among these catalysts compound **3** demonstrates better activity. Poor conversion was observed by running the polymerization in tetrahydrofuran (entry 7). We also introduced isopropyl alcohol or benzyl alcohol as initiators, 9-anthracenemethanol was identified as the best choice for this system after several polymerization trials in toluene at 50 °C (entries 3 and 8–11). To save reaction time, the reaction temperature was raised to 80 °C for examination of the effects on both living and immortal characteristics. Typical polymerization reactions were carried out at 80 °C in 5 mL toluene solution in the presence of 9-AnOH for l-lactide (LLA) using prescribed equivalent ratios of the monomers, catalysts (0.025 mmol) and 9-AnOH for the prescribed time. Representative results are collected in Table 1.

**Figure 4:**
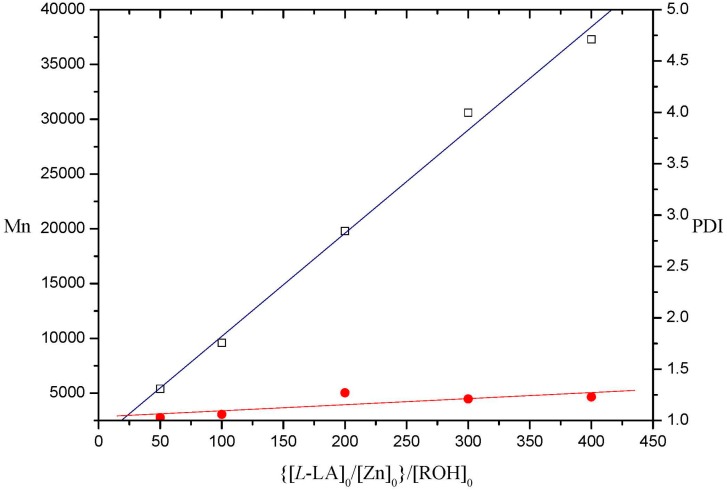
Polymerization of l-lactide catalyzed by **3** in toluene at 80 °C. (PDI= Mn/Mw in Table 2).

The linear relationship between the number-average molecular weight (Mn) and the monomer-to-initiator ratio ([M]_0_/[I]_0_) exhibited by **3** (entries 12–16) implies the “living” character of the polymerization process. That means the catalyst remains active for the subsequent addition of monomer. Representative results initiated by **3** are demonstrated in Figure 4.

**Table 2:** Polymerization of l-Lactide Using Compounds **1**–**7** as Catalysts in the Presence of 9-AnOH at 80 °C. *^a^*

Entry	Catalyst	{[L-LA]_0_:[Zn]_0_}:[9-AnOH]	Time (min)	Conv. (%) *^b^*	Mn(calcd) *^c^*	Mn(obsd) *^d^*	Mw/Mn *^d^*
1 *^e^*	**1**	50:1	30	65	5800	3700	1.09
2 *^e^*	**2**	50:1	30	25	1700	-	-
3 *^e^*	**3**	50:1	30	90	6700	4700	1.12
4 *^e^*	**4**	50:1	30	16	1600	-	-
5 *^e^*	**5**	50:1	30	5	600	-	-
6 *^e^*	**6**	50:1	30	43	3400	-	-
7 *^e,f^*	**3**	50:1	30	10	1000		
8 *^e^*	**3**	50:1(IPA)	30	90	6500	5800	1.12
9 *^e^*	**3**	50:1(BnOH)	30	67	4900	2700	1.09
10 *^e^*	**3**	150:1	120	93	20300	23300	1.06
11 *^e^*	**3**	150:1(IPA)	120	86	18600	22800	1.09
12	**3**	50:1	15	90	6700	5400	1.13
13	**3**	100:1	30	95	13900	9600	1.06
14	**3**	200:1	60	97	28000	19800	1.27
15	**3**	300:1	90	97	42200	30600	1.21
16	**3**	400:1	240	96	55600	37300	1.23
17	**3**	400:2	180	94	27300	20100	1.11
18	**3**	400:4	90	97	14200	13900	1.12
19	**3**	400:8	45	95	7100	7700	1.10
20	**3**	400:10	30	95	5700	6500	1.13
21	**3**	400:20	15	90	2800	3000	1.10
22	**3**	400:40	8	95	1600	1600	1.09
23	**7**	50:1	3	91	6700	5600	1.09
24	**7**	100:1	6	94	13700	11500	1.08
25	**7**	200:1	12	97	28100	22500	1.08
26	**7**	300:1	18	97	42100	32500	1.07
27	**7**	400:1	24	96	55500	38600	1.08
28	**7**	500:1	30	92	66500	45400	1.06
29	**7**	800:1	48	94	108600	57800	1.05
30	**7**	50:0	15	90	6600	26300	1.24

*^a^* 0.025 mmol catalyst in 5 mL toluene. *^b^* Obtained from ^1^H-NMR analysis. *^c^* Calculated from [M(lactide) × [M]_0_/[Zn]_0_ × conversion yield/([ROH]eq)] + M(ROH). *^d^* Obtained from GPC analysis times 0.58. *^e^* 50 °C. *^f^* in THF.

**Figure 5:**
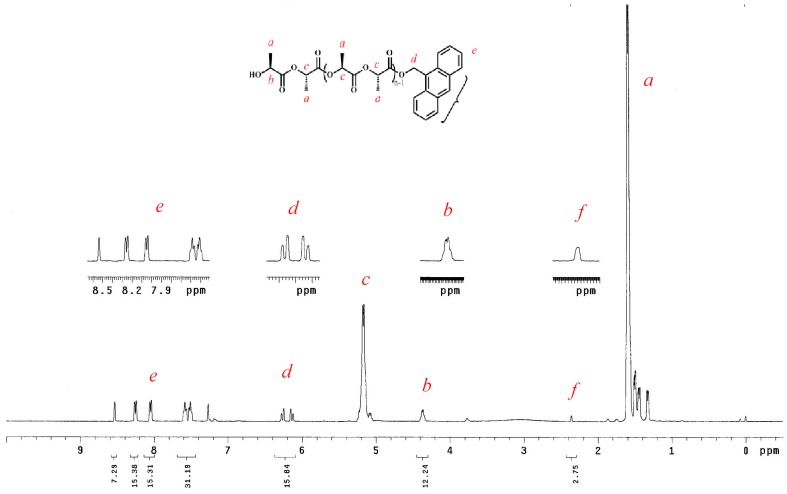
^1^H-NMR spectrum of PLLA-50 catalyzed by **3** in the presence of 9-AnOH in toluene.

The immortal character could be demonstrated with polymers formed in the presence of an excess of alcohol, which molecular weights could be predicted from the monomer-to-alcohol ratio. The ‘immortal’ character in this system was examined using different equiv. ratios (on [M]_0_/[Zn]_0_) of 9-AnOH as the chain transfer agent (entries 17–22) up to 400:40. The end group analysis is demonstrated by the ^1^H-NMR spectrum of the polymer produced from LLA and **3** ([M]_0_/[Zn]_0_ = 50), as shown in Figure 5. Peaks are similar to those found on the ^1^H-NMR spectra of polymers produced by aluminium benzotriazole phenoxide complexes [34], and are assignable to the corresponding protons in the proposed structure.

**Figure 6:**
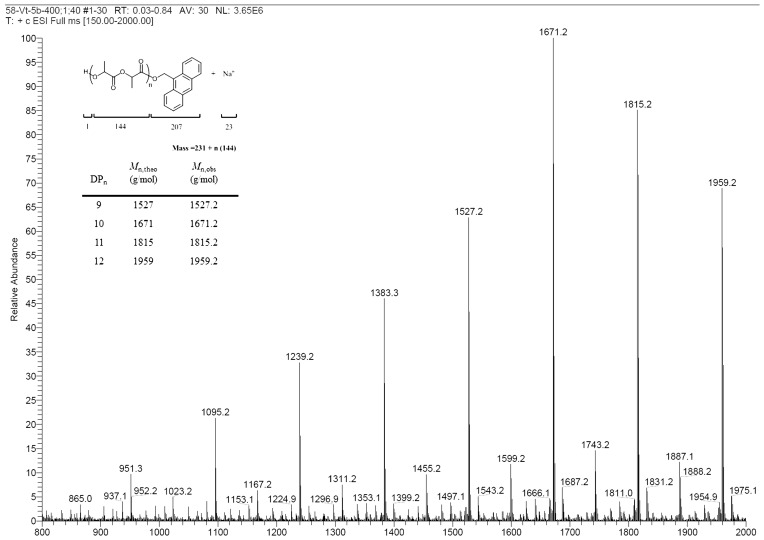
ESI-MS of 9-AnO-PLLA-H (Table 1, entry 22).

The ESI-MS analysis of a low molecular weight PLLA (*M*_n_(obsd) = 1600, Table 1, entry 22) clearly revealed a major population of PLLAs unequivocally confirmed as Na^+^ 9-AnO-PLA-H (Figure 6). The degree of polymerization indicated by this spectrum is in good agreement with the experimental value and the mass spectrum shows a cluster of homologous peaks separated by a molecular mass of ~144 Da corresponding to one lactide repeating unit. Based on those results, the metal alkoxide might form first, followed by the coordination-insertion mechanism [15,19].

**Figure 7:**
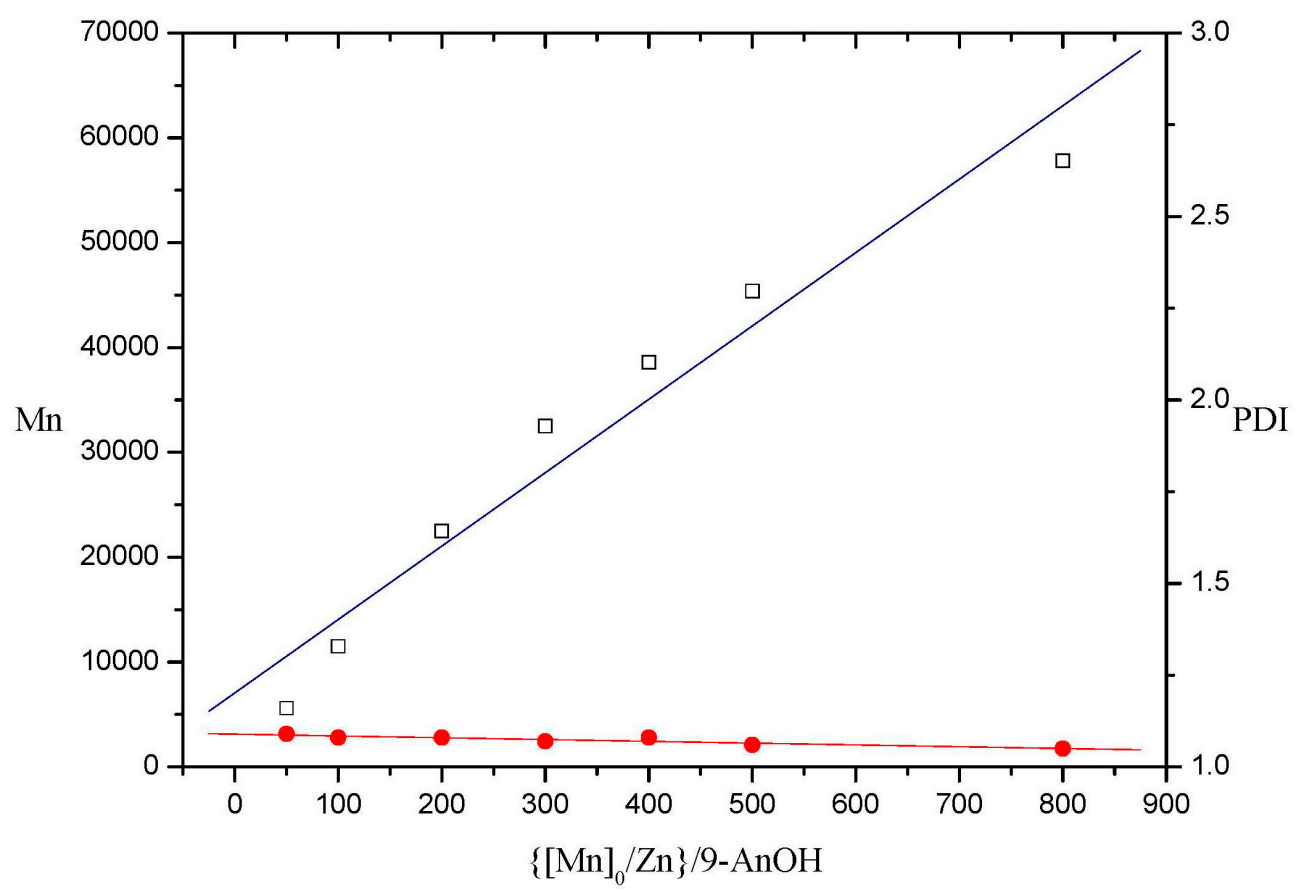
Polymerization of l-lactide catalyzed by **7** in toluene at 80 °C (PDI = Mn/Mw in Table 2).

Experimental results indicate that compound **7** exhibited better activities than compound **3** (entries 12 and 23) under the same conditions. These reactions gave PLLAs with increasing number-average molecular weight (Mn) and narrow PDIs values (1.05–1.09) upon increasing the mole-ratio up to 800:1. The plot of Mn *vs.* ([M]_0_/[I]_0_) demonstrated by those data initiated by **7** show the “living” character of the polymerization process, as shown in Figure 7 (entries 23–29). Compound **7** also demonstrated catalytic activity in the absence of alcohol. According to the molecular structure demonstrated by compound **1**, the polymerization catalyzed by **7** in the absence of alcohol might involve a coordination-insertion mechanism using amide group as initiator.

## 3. Experimental Section

### 3.1. General Information

All manipulations were carried out under an atmosphere of dinitrogen using standard Schlenk-line or drybox techniques. Solvents were refluxed over the appropriate drying agent and distilled prior to use. Deuterated solvents were dried over molecular sieves. ^1^H and ^13^C{^1^H} NMR spectra were recorded either on Varian Mercury-400 (400 MHz) or Varian Inova-600 (600 MHz) spectrometers in chloroform-*d* at ambient temperature unless stated otherwise and referenced internally to the residual solvent peak and reported as parts per million relative to tetramethylsilane. Elemental analyses were performed by Elementar Vario ELIV instrument. The GPC measurements were performed in THF at 35 °C with a Waters 1515 isocratic HPLC pump, a Waters 2414 refractive index detector, and a Waters Styragel column (HR4E). Molecular weights and molecular weight distributions were calculated using polystyrene as standard. The electrospray ionization mass spectrometry (ESI-MS) analyses were carried out with a Thermo Finnigan TSQ Quantum Triple Quadrupole Mass Spectrometer.

ZnEt_2_ (1.0 M in hexane, Sigma-Aldrich, St. Louis, MO, USA), 9-anthracenemethanol (Acros, Geel, Belgium), 2,4,6-trimethylaniline (Alfa Aesar, Ward Hill, MA, USA), triethylamine (TEDIA, Fairfield, OH, USA), 2-methoxyaniline (Acros), 2-(methylthio)aniline (Alfa Aesar), 2-methoxyethylamine (Acros), and propylamine (Acros) were used as supplied. Zn[(N(SiMe_3_)_2_]_2_ [35] and 4-chloro-3-coumarincarbaldehyde [36] were prepared by the literature method. Benzyl alcohol and 2-propanol were dried over calcium hydride and distilled before use. l-Lactide was recrystallized from toluene prior to use.

### 3.2. Preparations

*4-(mesitylamino)-2-oxo-2H-chromene-3-carbaldehyde.* To a flask containing 4-chloro-3-coumarincarbaldehyde (2.80 g, 10.0 mmol) in ethanol (40 mL) was added NEt_3_ (1.54 mL, 11.0 mmol) followed by the addition of 2,4,6-trimethylaniline (1.54 mL, 11.0 mmol) at room temperature. After 12 hours of stirring, all the volatiles were pumped off and the residue was extracted with toluene to afford a brown oil that was rinsed with hexane (5 mL) to afford a yellow solid. Yield, 2.24 g, 72.8%. ^1^H-NMR (400 MHz): δ 2.15 (s, 2,6-C*H_3_*, 6H), 2.38 (s, 4-C*H_3_*, 3H), 6.88 (m, C*H*-Ph, 1H), 7.00–7.022 (overlapped, C*H*-Ph and 3,5-C_6_*H_2_*, 3H), 7.31 (d, C*H*-Ph, 1H, *J =* 8.4 Hz), 7.52 (m, C*H*-Ph, 1H), 10.29 (s, C(=O)*H*, 1H), 13.10 (s, N*H*, 1H). ^13^C{^1^H}-NMR (100 MHz): δ 18.0 (2,6-*C*H_3_), 20.9 (4-*C*H_3_), 118.2, 123.8, 125.4, 129.7, 134.5 (*C*H-Ph), 96.3, 113.7, 133.2, 134.0, 138.5, 154.8, 158.4, 162.5 (*tert*-C), 192.2 (*C*(=O)H). Anal. Calc. for C_19_H_17_NO_3_: C, 74.25; H, 5.58; N, 4.56. Found: C, 74.12; H, 5.51; N, 4.39.

*L^1^H*. To a flask containing 4-(mesitylamino)-2-oxo-2H-chromene-3-carbaldehyde (1.54 g, 5.0 mmol) in ethanol (40 mL) 2,4,6-trimethylaniline (0.85 mL, 6.0 mmol) was added at room temperature. The reaction mixture was refluxed for 12 hours. All the volatiles were pumped off, and the residue was washed with hexane (10 mL) to afford a yellow solid. Yield, 1.60 g, 75%. ^1^H-NMR (400 MHz): δ 2.15 (s, 2,6-C*H_3_*, 6H), 2.17 (s, 2,6-C*H_3_*, 6H), 2.27 (s, 4-C*H_3_*, 3H), 2.35 (s, 4-C*H_3_*, 3H), 6.86 (m, C*H*-Ph, 1H), 6.88 (s, 3,5-C_6_*H_2_*, 2H), 6.97 (s, 3,5-C_6_*H_2_*, 2H), 7.07 (m, C*H*-Ph, 1H), 7.30 (m, C*H*-Ph, 1H), 7.45 (m, C*H*-Ph, 1H), 8.88 (s, C(=N)*H*, 1H), 13.09 (s, N*H*, 1H). ^13^C{^1^H}-NMR (100 MHz): δ 18.3 (2,6-*C*H_3_), 18.5 (2,6-*C*H_3_), 20.7 (4-*C*H_3_), 21.0 (4-*C*H_3_), 118.0, 123.5, 124.8, 128.8, 129.6, 132.9 (*C*H-Ph), 93.4, 115.3, 128.1, 133.7, 133.9, 135.4, 137.3, 146.4, 154.0, 156.0, 163.0 (*tert*-C), 163.1 (*C*(=N)H). Anal. Calc. for C_28_H_28_N_2_O_2_: C, 79.22; H, 6.65; N, 6.60. Found: C, 78.46; H, 6.22; N, 6.66.

*L^2^H.* The procedure for the preparation of **L^2^H** was similar to that used for **L^1^H** but with 4-(mesitylamino)-2-oxo-2*H*-chromene-3-carbaldehyde (0.46 g, 1.50 mmol), ethanol (40 mL) and 2-methoxyaniline (0.17 mL, 1.50 mmol). A yellowish-green solid was obtained. Yield, 0.86 g, 59%. ^1^H-NMR (400 MHz): δ 2.15 (s, 2,6-C*H_3_*, 6H), 2.36 (s, 4-C*H_3_*, 3H), 3.69 (s, OC*H_3_*, 3H), 6.84 (m, C*H*-Ph, 1H), 6.89 (m, C*H*-Ph, 1H), 6.97 (s, 3,5-C_6_*H_2_*, 2H), 6.98 (m, C*H*-Ph, 1H), 7.13 (m, C*H*-Ph, 1H), 7.15 (m, C*H*-Ph, 1H), 7.27 (m, C*H*-Ph, 1H), 7.33 (m, C*H*-Ph, 1H), 7.41 (m, C*H*-Ph, 1H), 9.21 (s, C(=N)*H*, 1H). ^13^C{^1^H}-NMR (100 MHz): δ 18.3 (2,6-*C*H_3_), 20.9 (4-*C*H_3_), 55.5 (O*C*H_3_), 111.4, 117.7, 118.0, 121.1, 123.5, 125.0, 126.6, 129.3, 132.6 (*C*H-Ph), 94.6, 116.1, 131.7, 135.7, 138.0, 151.9, 153.5, 155.2, 163.4 (*tert*-C), 155.8 (*C*(=N)H). Anal. Calc. for C_26_H_24_N_2_O_3_: C, 75.71; H, 5.86; N, 6.79. Found: C, 75.43; H, 5.99; N, 6.66. 

*L^3^H.* The procedure for the preparation of **L^3^H** was similar to that used for **L^1^H** but with 4-(mesitylamino)-2-oxo-2*H*-chromene-3-carbaldehyde (1.82 g, 5.92 mmol), ethanol (40 mL) and 2-(methylthio)aniline (0.90 mL, 7.2 mmol). A yellow solid was obtained. Yield, 1.12 g, 74%. ^1^H-NMR (400 MHz): δ 2.20 (s, 2,6-C*H_3_*, 6H), 2.32 (s, SC*H_3_*, 3H), 2.37 (s, 4-C*H_3_*, 3H), 6.86 (m, C*H*-Ph, 1H), 6.98 (s, 3,5-C_6_*H_2_*, 2H), 7.05 (m, C*H*-Ph, 1H), 7.14–7.20 (overlapped, C*H*-Ph, 3H), 7.28–7.32 (overlapped, C*H*-Ph, 2H), 7.45 (m, C*H*-Ph, 1H), 9.29 (s, C(=N)*H*, 1H), 13.09 (br, N*H*, 1H). ^13^C{^1^H}-NMR (100 MHz): δ 14.6 (S*C*H_3_), 18.5 (2,6-*C*H_3_), 21.1 (4-*C*H_3_), 117.1, 118.0, 123.6, 124.4, 124.9, 125.3, 126.5, 129.6, 133.0 (*C*H-Ph), 94.4, 115.1, 134.2, 134.3, 135.2, 137.5, 146.5, 153.9, 155.8, 163.1 (*tert*-C), 158.2 (*C*(=N)H). Anal. Calc. for C_26_H_24_N_2_O_2_S: C, 72.87; H, 5.64; N, 6.54. Found: C, 72.63; H, 5.37; N, 6.32.

*L^4^H.* The procedure for the preparation of **L^4^H** was similar to that used for **L^1^H** but with 4-(mesitylamino)-2-oxo-2*H*-chromene-3-carbaldehyde (1.54 g, 5.0 mmol), ethanol (40 mL) and propanamine (0.50 mL, 6.0 mmol). A yellow solid was obtained. Yield, 1.10 g, 63%. ^1^H-NMR (400 MHz): δ 0.94 (t, *J =* 7.6 Hz, CH_2_CH_2_C*H*_3_, 3H), 1.66 (sextet, *J =* 7.2 Hz, CH_2_C*H*_2_CH_3_, 2H), 2.05 (s, 2,6-C*H_3_*, 6H), 2.3 (s, 4-C*H_3_*, 3H), 3.48 (t, *J =* 6.8 Hz, C*H*_2_CH_2_CH_3_, 2H), 6.80 (m, C*H*-Ph, 1H), 6.95 (s, 3,5-C_6_*H_2_*, 2H), 7.00 (m, C*H*-Ph, 1H), 7.08 (m, C*H*-Ph, 1H), 7.37 (m, C*H*-Ph, 1H), 8.70 (s, C(=N)*H*, 1H), 13.60 (br, N*H*, 1H). ^13^C{^1^H}-NMR (100 MHz): δ 11.3 (*C*H_3_), 18.3 (2,6-*C*H_3_), 20.8 (4-*C*H_3_), 24.2 (*C*H_2_), 56.5 (*C*H_2_), 117.9, 123.3, 125.2, 129.2, 132.2 (*C*H-Ph), 92.6, 116.9, 129.8, 134.3, 140.8, 153.2, 155.1, 163.8 (*tert*-C), 160.2 (*C*(=N)H). Anal. Calc. for C_22_H_24_N_2_O_2_: C, 75.83; H, 6.94; N, 8.04. Found: C, 76.05; H, 7.05; N, 7.90.

*L^5^H.* The procedure for the preparation of **L^5^H** was similar to that used for **L^1^H** but with 4-(mesitylamino)-2-oxo-2*H*-chromene-3-carbaldehyde (1.54 g, 5.0 mmol), ethanol (40 mL) and 2-methoxylethylamine (0.53 mL, 6.0 mmol). A yellow solid was obtained. Yield, 1.13 g, 62%. ^1^H-NMR (400 MHz): δ 2.08 (s, 2,6-C*H_3_*, 6H), 2.35 (s, 4-C*H_3_*, 3H), 3.34 (s, OC*H*_3_, 3H), 3.57 (t, *J =* 5.6 Hz, C*H*_2_, 2H), 3.70 (t, *J =* 5.6 Hz, C*H*_2_, 2H), 6.81 m, C*H*-Ph, 1H), 6.96 (s, 3,5-C_6_*H_2_*, 2H), 7.07 (m, C*H*-Ph, 1H), 7.24 (m, C*H*-Ph, 1H), 7.38 (m, C*H*-Ph, 1H), 8.78 (s, C(=N)*H*, 1H), 13.76 (br, N*H*, 1H). ^13^C{^1^H}-NMR (100 MHz): δ 18.3 (2,6-*C*H_3_), 20.9 (4-*C*H_3_), 55.8 (*C*H_2_), 58.8 (O*C*H_3_), 72.1 (*C*H_2_), 117.9, 123.3, 125.0, 129.3, 132.3 (*C*H-Ph), 92.9, 116.4, 131.1, 135.2, 139.1, 153.3, 155.1, 163.5 (*tert*-C), 161.6 (*C*(=N)H). Anal. Calc. for C_22_H_24_N_2_O_3_: C, 72.50; H, 6.64; N, 7.69. Found: C, 71.95; H, 6.99; N, 7.63.

*L^6^H.* The procedure for the preparation of **L^6^H** was similar to that used for **L^1^H** but with 4-(mesitylamino)-2-oxo-2*H*-chromene-3-carbaldehyde (1.54 g, 5.0 mmol), ethanol (40 mL) and 2-(methylthio)ethanamine hydrochloride (0.76 g, 6.0 mmol). A yellow solid was obtained. Yield, 1.37 g, 72%. ^1^H-NMR (400 MHz): δ 2.10 (s, 2,6-C*H_3_*, 6H), 2.11 (s, SC*H*_3_, 3H), 2.35 (s, 4-C*H_3_*, 3H), 2.74 (t, *J* = 6.8 Hz, C*H*_2_, 2H), 3.76 (t, *J =* 6.8 Hz, C*H*_2_, 2H), 6.82 (t, *J =* 7.6 Hz, C*H*-Ph, 1H), 6.97 (s, 3,5-C_6_*H_2_*, 2H), 7.04 (d, *J =* 8.0 Hz, C*H*-Ph, 1H), 7.26 (d, *J =* 8.4 Hz, C*H*-Ph, 1H), 7.40 (t, *J =* 8.0 Hz, C*H*-Ph, 1H), 8.85 (s, C(=N)*H*, 1H). ^13^C{^1^H}-NMR (100 MHz): δ 15.6 (S*C*H_3_), 18.4 (2,6-*C*H_3_), 20.9 (4-*C*H_3_), 35.4 (*C*H_2_), 56.8 (*C*H_2_), 117.9, 123.4, 124.9, 129.4, 132.5 (*C*H-Ph), 92.9, 115.9, 132.3, 136.1, 137.6, 153.5, 155.3, 163.3 (*tert*-C), 161.7 (*C*(=N)H). Anal. Calc. for C_22_H_24_N_2_O_2_S: C, 69.44; H, 6.36; N, 7.36. Found: C, 69.97; H, 6.35; N, 7.60.

*L^1^ZnEt* (**1**). To a flask containing **L^1^H** (0.21 g, 0.50 mmol) in toluene (25 mL) ZnEt_2_ (1.0 M in hexane, 0.60 mL, 0.60 mmol) was added at 0 °C. The reaction mixture was allowed to warm to room temperature and reacted overnight. All the volatiles were pumped off, and the residue was washed with hexane (15 mL) to afford a yellow solid. Yield, 0.17 g, 66%. ^1^H-NMR (600 MHz): δ −0.12 (q, *J =* 7.8 Hz, C*H*_2_CH_3_, 2H), 0.59 (t, *J =* 8.4 Hz, CH_2_C*H*_3_, 3H), 2.08 (s, 2,6-C*H_3_*, 6H), 2.17 (s, 2,6-C*H_3_*, 6H), 2.28 (s, 4-C*H_3_*, 3H), 2.32 (s, 4-C*H_3_*, 3H), 6.74 (t, *J =* 7.8 Hz, C*H*-Ph, 1H), 6.89 (s, 3,5-C_6_*H_2_*, 2H), 6.92 (s, 3,5-C_6_*H_2_*, 2H), 7.04 (d, *J =* 8.4 Hz, C*H*-Ph, 1H), 7.13 (d, *J =* 7.8 Hz, C*H*-Ph, 1H), 7.36 (t, *J =* 7.2 Hz, C*H*-Ph, 1H), 8.83 (s, C(=N)*H*, 1H). ^13^C{^1^H}-NMR (150 MHz): δ −2.1 (*C*H_2_CH_3_), 11.1 (*C*H_2_CH_3_), 18.7 (2,6-*C*H_3_), 18.8 (2,6-*C*H_3_), 20.8 (4-*C*H_3_), 21.9 (4-*C*H_3_), 118.1, 123.2, 126.6, 129.2, 129.8, 132.6 (*C*H-Ph), 94.5, 117.7, 129.2, 134.7, 135.1, 144.8, 146.3, 153.1, 158.5, 165.3 (*tert*-C), 163.1 (*C*(=N)H). Anal. Calc. for C_30_H_32_N_2_O_2_Zn: C, 69.56; H, 6.23; N, 5.41. Found: C, 69.38; H, 6.31; N, 5.74.

*L^2^ZnEt* (**2**). The procedure for the preparation of **2** was similar to that used for **1** but with L^2^H (0.21 g, 0.50 mmol), toluene (25 mL) and ZnEt_2_ (1.0 M in hexane, 0.60 mL, 0.60 mmol). A yellowish-green solid was obtained. Yield, 0.18 g, 72%. ^1^H-NMR (600 MHz,):δ −0.13 (q, *J =* 7.8 Hz, C*H*_2_CH_3_, 2H), 0.62 (t, *J =* 8.4 Hz, CH_2_C*H*_3_, 3H), 2.09 (s, 2,6-C*H_3_*, 6H), 2.35 (s, 4-C*H_3_*, 3H), 3.85 (s, OC*H_3_*, 3H), 6.74 (m, C*H*-Ph, 1H), 6.92 (d, *J =* 7.8Hz, C*H*-Ph, 1H), 6.95 (s, 3,5-C_6_*H_2_*, 2H), 7.02–7.05 (overlapped, C*H*-Ph, 2H), 7.20–7.23 (overlapped, C*H*-Ph, 2H), 7.35–7.37 (overlapped, C*H*-Ph, 2H), 9.15 (s, C(=N)*H*, 1H). ^13^C{^1^H}-NMR (150 MHz,): δ −2.3 (*C*H_2_CH_3_), 11.3 (CH_2_*C*H_3_), 18.7 (2,6-*C*H_3_), 21.0 (4-*C*H_3_), 55.6 (O*C*H_3_), 110.6, 118.0, 121.1, 121.7, 123.1, 126.6, 127.0, 129.7, 132.5 (*C*H-Ph), 95.2, 117.8, 129.2, 134.6, 139.3, 144.9, 151.0, 153.2, 157.5, 165.3 (*tert*-C), 163.6 (*C*(=N)H). Anal. Calc. for C_28_H_28_N_2_O_3_Zn: C, 66.47; H, 5.58; N, 5.54. Found: C, 65.87; H, 5.70; N, 5.25. 

*L^3^ZnEt* (**3**). The procedure for the preparation of **3** was similar to that used for **1** but with L^3^H (0.22 g, 0.50 mmol), toluene (25 mL), and ZnEt_2_ (1.0 M in hexane, 0.60 mL, 0.60 mmol). A yellow solid was obtained. Yield, 0.19 g, 73%. ^1^H-NMR (600 MHz): δ −0.15(q, *J =* 7.8 Hz, C*H*_2_CH_3_, 2H), 0.60 (t, *J =* 8.4 Hz, CH_2_C*H*_3_, 3H), 2.12 (s, 2,6-C*H_3_*, 6H), 2.34 (s, 4-C*H_3_*, 3H), 2.43 (s, SC*H_3_*, 3H), 6.73 (m, C*H*-Ph, 1H), 6.94 (s, 3,5-C_6_*H_2_*, 2H), 7.05 (m, C*H*-Ph, 1H), 7.22–7.27 (overlapped, C*H*-Ph, 2H), 7.35–7.42 (overlap, C*H*-Ph, 4H), 9.10(s, C(=N)*H*, 1H). ^13^C{^1^H}-NMR (150 MHz): δ −1.8 (*C*H_2_CH_3_), 11.5 (CH_2_*C*H_3_), 17.7 (S*C*H_3_), 18.7 (2,6-*C*H_3_), 21.0 (4-*C*H_3_), 118.1, 120.9, 123.1, 126.8, 128.7, 129.3, 129.8, 132.6 (*C*H-Ph), 95.2, 117.8, 129.4, 130.2, 134.6, 145.0, 149.4, 153.3, 158.2, 165.1 (*tert*-C), 163.9 (*C*(=N)H). Anal. Calc. for C_28_H_28_N_2_O_2_SZn: C, 64.43; H, 5.41; N, 5.37. Found: C, 64.85; H, 5.88; N, 5.69.

*L^4^ZnEt* (**4**). The procedure for the preparation of **4** was similar to that used for **1** but with L^4^H (0.19 g, 0.50 mmol), toluene (25 mL) and ZnEt_2_ (1.0 M in hexane, 0.60 mL 0.60 mmol). A pale-yellow solid was obtained. Yield, 0.14 g, 63%. ^1^H-NMR (600 MHz): δ 0.01 (q, *J =* 7.8Hz, C*H*_2_CH_3_, 2H), 0.83 (t, *J =* 7.8 Hz, CH_2_C*H*_3_, 3H), 0.95 (t, *J =* 7.2 Hz, CH_2_CH_2_C*H*_3_, 3H), 1.72 (sextet, *J =* 7.2 Hz, CH_2_C*H*_2_CH_3_, 2H), 2.02 (s, 2,6-C*H_3_*, 6H), 2.32 (s, 4-C*H_3_*, 3H), 3.59 (t, *J =* 6.6 Hz, C*H*_2_CH_2_CH_3_, 2H), 6.72 (t, *J =* 7.8 Hz, C*H*-Ph, 1H), 6.92 (s, 3,5-C_6_*H_2_*, 2H), 6.98 (d, *J =* 9.0 Hz, C*H*-Ph, 1H), 7.19 (d, *J =* 7.2 Hz, C*H*-Ph, 1H), 7.34 (t, *J =* 7.2Hz, C*H*-Ph, 1H), 8.93 (s, C(=N)*H*, 1H). ^13^C{^1^H}-NMR (150 MHz): δ −2.1 (*C*H_2_CH_3_), 11.3, 11.4 (CH_2_*C*H_3_ and CH_2_CH_2_*C*H_3_), 18.8 (2,6-*C*H_3_), 20.9 (4-*C*H_3_), 25.4 (*C*H_2_), 63.0 (*C*H_2_), 117.9, 123.2, 126.4, 129.7, 132.3 (*C*H-Ph), 93.5, 117.8, 129.3, 134.6, 144.9, 152.9, 158.1, 167.5 (*tert*-C), 166.0 (*C*(=N)H). Anal. Calc. for C_24_H_28_N_2_O_2_Zn: C, 65.24; H, 6.39; N, 6.34. Found: C, 65.13; H, 6.37; N, 6.33. 

*L^5^ZnEt* (**5**). The procedure for the preparation of **5** was similar to that used for **1** but with L^5^H (0.183 g, 0.50 mmol), toluene (25 mL) and ZnEt_2_ (1.0 M in hexane, 0.60 mL, 0.60 mmol). A pale-yellow solid was obtained. Yield, 0.12 g, 52%. ^1^H-NMR (600 MHz): δ −0.10 (q, *J =* 7.8 Hz, C*H*_2_CH_3_, 2H), 0.75 (t, *J =* 7.8 Hz, CH_2_C*H*_3_, 3H), 1.96 (s, 2,6-C*H_3_*, 6H), 2.25 (s, 4-C*H_3_*, 3H), 3.28 (s, OC*H*_3_, 3H), 3.51 (t, *J =* 5.4 Hz, C*H*_2_, 2H), 3.70 (t, *J =* 5.4 Hz, C*H*_2_, 2H), 6.64 (m, C*H*-Ph, 1H), 6.84 (s, 3,5-C_6_*H_2_*, 2H), 6.90 (m, C*H*-Ph, 1H), 7.12 (m, C*H*-Ph, 1H), 7.26 (m, C*H*-Ph, 1H), 8.79 (s, C(=N)*H*, 1H). ^13^C{^1^H}-NMR (150 MHz): δ −2.3 (*C*H_2_CH_3_), 11.6 (CH_2_*C*H_3_), 18.6 (2,6-*C*H_3_), 20.9 (4-*C*H_3_), 58.9 (O*C*H_3_), 60.2 (*C*H_2_), 72.4 (*C*H_2_), 117.9, 123.3, 125.0, 129.3, 132.3 (*C*H-Ph), 93.8, 118.0, 129.2, 134.3, 145.0, 152.9, 157.6, 166.0 (*tert*-C), 166.4 (*C*(=N)H). Anal. Calc. for C_24_H_28_N_2_O_3_Zn: C, 62.96; H, 6.16; N, 6.12. Found: C, 62.70; H, 5.90; N, 5.61.

*L^6^ZnEt* (**6**). The procedure for the preparation of **6** was similar to that used for **1** but with L^6^H (0.19 g, 0.50 mmol), toluene (25 mL) and ZnEt_2_ (1.0 M in hexane, 0.60 mL, 0.60 mmol). A yellow solid was obtained. Yield, 0.16 g, 67%. ^1^H-NMR (600 MHz): δ-0.02 (q, *J =* 8.4 Hz, C*H*_2_CH_3_, 2H), 0.74 (t, *J =* 7.8 Hz, CH_2_C*H*_3_, 3H), 2.04 (s, 2,6-C*H_3_*, 6H), 2.13 (s, SC*H*_3_, 3H), 2.33 (s, 4-C*H_3_*, 3H), 2.77 (t, *J =* 6.0 Hz, C*H*_2_, 2H), 3.83 (t, *J =* 6.6 Hz, C*H*_2_, 2H), 6.71 (m, C*H*-Ph, 1H), 6.92 (s, 3,5-C_6_*H_2_*, 2H), 6.99 (m, C*H*-Ph, 1H), 7.20 (m, C*H*-Ph, 1H), 7.34 (m, C*H*-Ph, 1H), 8.95 (s, C(=N)*H*, 1H). ^13^C{^1^H}-NMR (150 MHz): δ −2.2 (*C*H_2_CH_3_), 11.6 (CH_2_*C*H_3_), 15.2 (S*C*H_3_), 18.8 (2,6-*C*H_3_), 20.9 (4-*C*H_3_), 36.8 (*C*H_2_), 57.1 (*C*H_2_), 118.0, 123.0, 126.6, 129.7, 132.3 (*C*H-Ph), 93.8, 117.9, 129.4, 134.5, 145.1, 153.1, 157.9, 165.4 (*tert*-C), 166.3 (*C*(=N)H). Anal. Calc. for C_24_H_28_N_2_O_2_SZn: C, 60.82; H, 5.95; N, 5.91. Found: C, 61.32; H, 6.24; N, 5.97.

*L^3^ZnN(SiMe_3_)_2_* (**7**). A solution of Zn[N(SiMe_3_)_2_]_2_ (0.23 g, 0.6 mmol) in toluene (10 mL) was added dropwise at 0 °C to a flask containing L^3^H (0.22 g, 0.50 mmol) in toluene (10 mL). The reaction mixture was allowed to warm to room temperature and reacted overnight. All the volatiles were pumped off, and the residue was washed with hexane (15 mL) to afford a yellow solid. Yield, 0.18 g, 58%. ^1^H-NMR (600 MHz): δ ‒0.43 (s, N(Si(C*H*_3_)_3_)_2_, 18H), 2.14 (s, 2,6-C*H_3_*, 6H), 2.29 (s, 4-C*H_3_*, 3H), 2.40 (s, SC*H_3_*, 3H), 6.66 (t, *J =* 7.8 Hz, C*H*-Ph, 1H), 6.86 (d, *J =* 8.4 Hz, C*H*-Ph, 1H), 6.92 (s, 3,5-C_6_*H_2_*, 2H), 7.17 (d, *J =* 8.4 Hz, C*H*-Ph, 1H), 7.23 (t, *J =* 7.2 Hz, C*H*-Ph, 1H), 7.31 (t, *J =* 7.8 Hz, C*H*-Ph, 1H), 7.35 (t, *J =* 7.8 Hz, C*H*-Ph, 1H), 7.40 (overlapped, C*H*-Ph, 2H), 9.15 (s, C(=N)*H*, 1H). ^13^C{^1^H}-NMR (150 MHz): δ 4.4 (N(Si(*C*H_3_)_3_)_2_), 18.6 (S*C*H_3_), 18.9 (2,6-*C*H_3_), 20.9 (4-*C*H_3_), 118.2, 120.5, 123.2, 127.0, 127.1, 129.3, 129.7, 130.4, 132.8 (*C*H-Ph), 95.0, 117.8, 129.2, 130.4, 135.5, 144.2, 148.3, 153.3, 159.8, 164.8 (*tert*-C), 164.1 (*C*(=N)H). Anal. Calc. for C_32_H_41_N_3_O_2_SSi_2_Zn: C, 58.83; H, 6.33; N, 6.43. Found: C, 57.90; H, 6.05; N, 6.14.

*Procedure for Polymerization of l-Lactide.* Typically, to a flask containing a prescribed amount of l-lactide, 9-AnOH and catalyst toluene (5 mL) was added. The reaction mixture was stirred at 50 °C or 80 °C for the prescribed time. After the reaction was quenched by the addition of acetic acid solution (10 mL, 0.35 M), the resulting mixture was poured into *n*-hexane (25 mL) to precipitate polymers. Crude products were recrystallized from THF/hexane and dried *in vacuo* to a constant weight.

### 3.3. Crystal Structure Data

Crystals were grown from toluene/hexane (compound **1**) or concentrated hexane solution (compound **7**), and isolated by filtration. Suitable crystals of **1** or **7** were mounted onto Mounted CryoLoop (Hampton Research, Aliso Viejo, CA, USA; size: 0.5–0.7 mm) using perfluoropolyether oil (FOMBLIN^®^Y, Aldrich) and cooled rapidly in a stream of cold nitrogen gas using an Oxford Cryosystems Cryostream unit. Diffraction data were collected at 100 K using an Oxford Gemini S diffractometer. Empirical absorption correction was based on spherical harmonics, implemented in the SCALE3 ABSPACK scaling algorithm from CrysAlis RED (Oxford Diffraction Ltd., Abingdon, UK). The space group determination was based on a check of the Laue symmetry and systematic absences and was confirmed using the structure solution. The structure was solved by direct methods using a SHELXTL package [37]. All non-H atoms were located from successive Fourier maps, and hydrogen atoms were refined using a riding model. Anisotropic thermal parameters were used for all non-H atoms, and fixed isotropic parameters were used for H atoms. Some details of the data collection and refinement are given in Table 3.

**Table 3:** Summary of crystal data for compounds **1** and **7**.

	1	7
Formula	C_30_H_32_N_2_O_2_Zn	C_32_H_41_N_3_O_2_SSi_2_Zn
Fw	517.95	653.29
T, K	150(2)	150(2)
Crystal system	Orthorhomic	Triclinic
Space group	*P2_1_2_1_2_1_*	*P-1*
*a*, Å	9.4413(2)	10.1352(3)
*b*, Å	12.7962(2)	11.1435(5)
*c*, Å	22.3768(4)	14.9829(5)
*α*°	90	78.698(3)
*β*°	90	82.913(3)
*γ*°	90	89.969(3)
*V*, Å^3^	2703.40(9)	1646.21(10)
Z	4	2
*ρ*_calc_, Mg/m^3^	1.273	1.318
*μ* (Mo Kα), mm^−1^	0.936	0.915
Reflections collected	29922	18473
No. of parameters	316	370
*R1 ^a^*	0.0328	0.0420
w *R2 ^a^*	0.0912	0.1255
GoF *^b^*	1.004	1.000

*^a^* *R1* =[ Σ|F_0_| − |F_c_|]/Σ |F_0_|]; w*R*2 = [Σ w(F_0_^2^ − F_c_^2^)^2^/Σ w(F_0_^2^)^2^]^1/2^, w = 0.10. *^b^* GoF = [Σ*w*(F_0_^2^ − F_c_^2^)^2^/(*N*_rflns_ − *N*_params_)]^1/2^.

CCDC reference numbers 1045465–1045466 (for **1** and **7**, respectively) contain the supplementary crystallographic data for this paper. These data can be obtained free of charge from The Cambridge Crystallographic Data Centre via www.ccdc.cam.ac.uk/data_request/cif.

## 4. Conclusions

A series of zinc complexes containing coumarin-based anilido-aldimine ligands has been prepared and fully characterized. Based on the molecular structures demonstrated by **1** and **7**, the metal center adopts a distorted tetrahedral geometry with the coumarin-based anilido-aldimine ligands. Coordination of the carbonyl group from another molecule happens in the case of ligands without pendant functionalities, resulting in the formation of a coordination polymer. This phenomenon also supports the expected coordination to the metal center from the carbonyl group of monomers. Under optimized condition, compound **3** and **7** demonstrate efficient activities for the controlled polymerization of LLA with both living and immortal characteristics. Preliminary studies on fine-tuning of ligand precursors and further application of metal complexes to the catalytic reactions are currently underway.

## References

[1] Uhrich K.E., Cannizzaro S.M., Langer R.S., Shakesheff K.M. (1999). Polymeric systems for controlled drug release. Chem. Rev..

[2] Dechy-Cabaret O., Martin-Vaca B., Bourissou D. (2004). Controlled ring-opening polymerization of lactide and glycolide. Chem. Rev..

[3] Williams C.K. (2007). Synthesis of functionalized biodegradable polyesters. Chem. Soc. Rev..

[4] Tong R., Cheng J. (2008). Paclitaxel-initiated, controlled polymerization of lactide for the formulation of polymeric nanoparticulate delivery vehicles. Angew. Chem. Int. Ed..

[5] O’Keefe B.J., Hillmyer M.A., Tolman W.B. (2001). Polymerization of lactide and related cyclic esters by discrete metal complexes. J. Chem. Soc. Dalton Trans..

[6] Wu J., Yu T.L., Chen C.T., Lin C.C. (2006). Recent developments in main group metal complexes catalyzed/initiated polymerization of lactides and related cyclic esters. Coord. Chem. Rev..

[7] Platel R.H., Hodgson L.M., Williams C.K. (2008). Biocompatible Initiators for Lactide Polymerization. Polym. Rev..

[8] Wheaton C.A., Hayes P.G., Ireland B.J. (2009). Complexes of Mg, Ca and Zn as homogeneous catalysts for lactide polymerization. Dalton Trans..

[9] Stanford M.J., Dove A.P. (2010). Stereocontrolled ring-opening polymerisation of lactide. Chem. Soc. Rev..

[10] Sutar A.K., Maharana T., Dutta S., Chen C.T., Lin C.C. (2010). Ring-opening polymerization by lithium catalysts: an overview. Chem. Soc. Rev..

[11] Ajellal N., Carpentier J.F., Guillaume C., Guillaume S.M., Helou M., Poirier V., Sarazin Y., Trifonov A. (2010). Metal-catalyzed immortal ring-opening polymerization of lactones, lactides and cyclic carbonates. Dalton Trans..

[12] Doyle D.J., Gibson V.C., White A.J.P. (2007). Synthesis and structures of bimetallic and polymeric zinc coordination compounds supported by salicylaldiminato and anilido–aldimine ligands. Dalton Trans..

[13] Shang X., Liu X., Cui D. (2007). Yttrium bis(alkyl) and bis(amido) complexes bearing N,O-multidentate ligands. Synthesis and catalytic activity towards ring-opening polymerization of l-lactide. J. Polym. Sci. Part A: Polym. Chem..

[14] Gao W., Cui D., Liu X., Zhang Y., Mu Y. (2008). Rare-earth metal bis(alkyl)s supported by a quinolinyl anilido-imine ligand: synthesis and catalysis on living polymerization of *ε*-caprolactone. Organometallics.

[15] Yao W., Mu Y., Gao A., Gao W., Ye L. (2008). Bimetallic anilido-aldimine Al or Zn complexes for efficient ring-opening polymerization of ε-caprolactone. Dalton Trans..

[16] Tsai Y.H., Lin C.H., Lin C.C., Ko B.T. (2009). Tridentate anilido-aldimine magnesium and zinc complexes as efficient catalysts for ring-opening polymerization of ε-caprolactone and l-lactide. J. Polym. Sci. Part A: Polym. Chem..

[17] Liu Y.C., Lin C.H, Ko B.T., Ho R.M. (2010). Ring-opening polymerization of β-butyrolactone catalyzed by efficient magnesium and zinc complexes derived from tridentate anilido-aldimine ligand. J. Polym. Sci. Part A Polym. Chem..

[18] Allan L.E.N., Bélanger J.A., Callaghan L.M., Cameron D.J.A., Decken A., Shaver M.P. (2012). Anilido-aldimine aluminum complexes: Synthesis, characterization and lactide polymerization. J. Organomet. Chem..

[19] Wang C.H., Li C.Y., Huang B.H., Lin C.C., Ko B.T. (2013). Synthesis and structural determination of zinc complexes based on an anilido-aldimine ligand containing an O-donor pendant arm: Zinc alkoxide derivative as an efficient initiator for ring-opening polymerization of cyclic esters. Dalton Trans..

[20] Lacy A., O’Kennedy R. (2004). Studies on coumarins and coumarin-related compounds to determine their therapeutic role in the treatment of cancer. Curr. Pharm. Des..

[21] Kulkarni M.V., Kulkarni G.M., Lin C.H., Sun C.M. (2006). Recent advances in coumarins and 1-azacoumarins as versatile biodynamic agents. Curr. Med. Chem..

[22] Musa M.A., Cooperwood J.S., Khan M.O.F. (2008). A review of coumarin derivatives in pharmacotherapy of breast cancer. Curr. Med. Chem..

[23] Grazul M., Budzisz E. (2009). Biological activity of metal ions complexes of chromones, coumarins and flavones. Coord. Chem. Rev..

[24] Wagner B.D. (2009). The use of coumarins as environmentally-sensitive fluorescent probes of heterogeneous inclusion systems. Molecules.

[25] Hagfeldt A., Boschloo G., Sun L., Kloo L., Pettersson H. (2010). Dye-sensitized solar cells. Chem. Rev..

[26] Kricheldorf H.R., Berl M., Scharnagl N. (1988). Poly(1actones). 9. Polymerization mechanism of metal alkoxide initiated polymerizations of lactide and various lactones. Macromolecules.

[27] Dubois P., Jacobs C., Jérôme R., Teyssié P. (1991). Macromolecular engineering of polylactones and polylactides. 4. Mechanism and kinetics of lactide homopolymerization by aluminum isopropoxide. Macromolecules.

[28] Silvernail C.M., Yao L.J., Hill L.M. R., Hillmyer M.A., Tolman W.B. (2007). Structural and mechanistic studies of bis(phenolato)amine zinc(II) catalysts for the polymerization of ε-caprolactone. Inorg. Chem..

[29] Chen C.T., Chan C.Y., Huang C.A., Chen M.T., Peng K.F. (2007). Zinc anilido-oxazolinate complexes as initiators for ring opening polymerization. Dalton Trans..

[30] Chen M.T., Chen C.T. (2011). Structural and catalytic studies of zinc complexes containing amido-oxazolinate ligands. Dalton Trans..

[31] Dove A.P., Gibson V.C., Marshall E.L., White A.J. P., Williams D.J. (2004). Magnesium and zinc complexes of a potentially tridentate β-diketiminate ligand. Dalton Trans..

[32] Abbina S., Du G. (2012). Chiral amido-oxazolinate zinc complexes for asymmetric alternating copolymerization of CO_2_ and cyclohexene oxide. Organometallics.

[33] Dickson R.S., Fallon G.D., Zhang Q.Q. (2000). Dimeric diphenylzinc adducts with cyclic thioethers. J. Chem. Soc. Dalton Trans..

[34] Li C.Y., Tsai C.Y., Lin C.H., Ko B.T. (2011). Synthesis, structural characterization and reactivity of aluminium complexes supported by benzotriazole phenoxide ligands: Air-stable alumoxane as an efficient catalyst for ring-opening polymerization of l-lactide. Dalton Trans..

[35] Bochmann M., Bwembya G., Webb K.J., Malik M.A., Walsh J.R., O’Brien P. (1997). Arene chalcogenolato complexes of zinc and cadmium. Inorg. Synth..

[36] Sabatié A., Végh D., Loupy A., Floch L. (2001). Synthesis of aromatic and heteroaromatic annelated [1,4]diazepines. ARKIVOC.

[37] Sheldrick G.M. (1997). SHELXTL-97, Program for Refinement of Crystal Structures.

